# Development and psychometric testing of the Spanish version of the Caregiver Preparedness Scale

**DOI:** 10.1002/nop2.732

**Published:** 2020-12-19

**Authors:** Belen Gutierrez‐Baena, Carmen Romero‐Grimaldi

**Affiliations:** ^1^ Vocational training center “María de Madariaga” Cádiz Spain; ^2^ Hospital “Viamed Bahía de Cádiz,” Chiclana de la Frontera Cádiz Spain; ^3^ Nursing Faculty “Salus Infirmorum” University of Cadiz Cádiz Spain; ^4^ Centro de Investigación Biomédica en Red de Salud Mental (CIBERSAM), Instituto de Salud Carlos III. Madrid Spain

**Keywords:** caregiver preparedness scale, caregivers, instrument development, nursing, preparedness, psychometrics, reliability, Spanish version, validity

## Abstract

**Aim:**

To psychometrically test the Spanish version of the Caregiver Preparedness Scale (CPS) and document the preparedness level of caregivers.

**Design:**

A descriptive and validation study.

**Method:**

Purposive sampling method was used to select 171 family caregivers Spain. The scale was cross‐culturally adapted through a process that included translation, comparison with versions in other languages and back‐translation, review, pre‐testing and validity, and reliability tests.

**Results:**

The Spanish family caregivers are mainly female (79%) and married (75%). The Spanish version of the CPS presents changes with respect to the original. Confirmatory factor analysis supported the single‐factor model. Analysis of internal consistency yielded a Cronbach's *α* of 0.89. Significant correlations (*p* < .01) with other scales supported convergent validity. A descriptive analysis of the validated scale showed average levels of preparation (2.16 out of 4). Caregivers felt better prepared to attend to the patient's physical needs than emotional or spiritual needs.

## 1 INTRODUCTION

Family caregiving researchers have explored negative consequences of care such as caregiver stress, burden or strain. However, the caregiving quality, relationship and preparedness for caregiving have received little attention as effect moderators. The CPS was designed to measure the caregiver preparedness at home (Archbold et al., 1990) and has been demonstrated to be an instrument with excellent psychometric properties (Schumacher et al., 2007). The creation of this scale was intended to address a gap in the literature on influence of mutuality and preparedness on the caregiver's role.

### 1.1 Background

It is estimated that 349 million people worldwide are care‐dependent, of whom 101 million (29%) are over 60 years of age (World Health Organization, 2015). According to the National Statistics Institute, in Spain there are more than two million dependent people: 29.93% of the population over 64 years of age is in a situation of dependency, of whom 84% need assistance with their daily hygiene and 63% with their food intake. In addition, 89.4% of people who need care receive informal support (Abellán et al., 2011, Roguero‐García, 2009). Dependence on care is defined as the need for help or frequent human care beyond what a healthy adult usually requires and also involves assistance in carrying out daily activities (Delicado et al., 2004). In older people, coexisting chronic diseases (multimorbidities) are frequently associated with the need for medical and social care (Fortin et al., 2007). The existing policies in our country are based on maintaining these people permanently at home as long as possible, emphasizing this as a more humane and less costly response and they also reveal the family as the habitual core of coexistence (Bódalo, 2010).

In many cases, the caregiver lacks the necessary training to look after another person. Furthermore, in Spain as in other countries, there is no specific theoretical–practical training. Situations such as not knowing the evolution of a specific disease, feeling alone and physical and emotional fatigue are indicated to be the main drawbacks suffered by caregivers at work (López et al., 2009). In relation to caregiver training and work overload, greater preparation has been described as associated with better mental health and less stress on the caregiving role even when the demand for caregiving is high (Yang et al., 2014). On the other hand, the caregiver's insufficient preparation decreases the quality of the relationship and increases the stress experienced by the caregiver (Schumacher et al., 2007). Therefore, by detecting the possible shortcomings of the caregiver, we would be able to design interventions that increase the training of the caregiver, while reducing periods of work overload and increasing the quality of the care provided.

In our context, there are validated instruments to measure different aspects of the circumstances of family caregivers such as work overload, resilience and quality of life (Crespo et al., 2014; Garratt et al., 1993; Vélez et al., 2012). However, the ability to apply caregiving is rarely evaluated with quantitative methods. An instrument to evaluate the training of a caregiver is the Caregiver Preparedness Scale (CPS), created by the research group of Patricia G. Archbold in the USA to measure the level of preparation of the caregivers. In its initial validation, it presented reliability values of 0.86 (Archbold et al., 1990). Subsequently, different versions of the scale were tested, which also demonstrated optimal internal consistency values of 0.90, 0.94 and 0.88 (Henriksson et al., 2012; Pucciarelli et al., 2014; Ugur et al., 2017, respectively). Archbold's exploratory factor analysis (EFA) with 78 caregivers explained 50% of the variance and established a single factor (Archbold et al., 1990). Through the AFC, Hudson supported the existence of a single factor with 106 Australian caregivers that explained 66.7% of the variance (Hudson & Hayman‐White, 2006). Subsequently, Henriksson et al. and Pucciarelli et al. corroborated the one‐dimensional aspect of the scale with samples of 125 and 156 caregivers, respectively (Henriksson et al., 2012; Pucciarelli et al., 2014). Adapted versions of the original scale and the original scale itself have been used to assess the ability of caregivers of patients with cancer (Hudson & Hayman‐White, 2006), Parkinson's disease (Carter et al., 2010), strokes (Pucciarelli et al., 2014), cardiac surgery (Kneeshaw et al., 1999), life‐threatening diseases (Henriksson et al., 2012) and heart failure (Petruzzo et al., 2017). Finally, its usefulness has been demonstrated in the distinction between well‐prepared caregivers and those who are not (Henriksson et al., 2015). In Spain, some studies have addressed the quality of life and burden of caregivers; however, there are no validated instruments to measure the level of preparation for caregiving.

## 2 THE STUDY

### 2.1 Aim

The purpose of this study was to cross‐culturally adapt the CPS to the Spanish language and assess its psychometric properties on a sample of Spanish family caregivers and document the level of preparedness to provide care to dependent individuals.

### 2.2 Design

A cross‐sectional design was employed, and a psychometric validation study was developed.

### 2.3 Methodology

Data were collected between November 2018–June 2019 in Cadiz (Spain). The phases of the study were as follows: (a) cross‐cultural adaptation process, usability testing and pre‐testing; (b) psychometric evaluation (internal consistency, factorial analysis, item analysis and validity); and (c) descriptive analysis of the validated scale.

#### 2.3.1 Phase 1: cross‐cultural adaptation process, usability testing and pre‐testing

The original version in English (Archbold et al., 1990) was translated into Spanish by two independent translators. It is recommended that one translator knows the objectives of the study so as to offer reliability in the intended measurement and the other translator does not know about these objectives to elicit unexpected meanings from the original scale (Guillemin et al., 1992; Hendricson et al., 1989). Therefore, one translation was done by a multilingual RN expert in nursing methodology who was informed of the objectives of the original investigation and the other by a professional translator without knowledge of nursing and the objectives of the study. The two direct translations were discussed jointly by the researchers to detect errors and divergent interpretations of items (Escobar‐Bravo, 2004) and obtain a definitive version of the translations. The procedure is recommended for using cross‐cultural adaptation of scales in different countries and languages (Beaton et al., 2000).

A qualitative study was used to investigate the usability of the Spanish version of the CPS and how the caregivers perceived and interpreted the questions before distributing the Spanish CPS. Five caregivers who agreed to participate in the usability test were interviewed in a private room in the hospital for about 30 min. The transcribed interviews were analyzed and put in the following categories: “social help, home care, religiosity and family.” (Gutiérrez & Romero‐Grimaldi, 2018). The qualitative data were collected between September 2017–May 2018.

The final version was checked using a pre‐test technique to detect errors and deviations in the translation (Guillemin et al., 1993) and ensure that the language used suited the target population of the scale (Escobar‐Bravo, 2004). Ten individuals who met the eligibility criteria were asked to identify any ambiguous or difficult‐to‐understand items and examine the instructions to finalize the scale. The Spanish Version of the Caregiver Preparedness Scale obtained through the above procedure is referred to in this study as S‐CPS.

#### 2.3.2 Phase 2: psychometric evaluation

SPSS 21.0 and AMOS 26.0 were used for data analysis. For all analyses, a *p*‐value less than 0.05 was considered statistically significant. The sociodemographic characteristics of the sample were analyzed using frequency analysis. Kendall W analysis was used to measure content validity. Values of this index close to or equal to 1 are interpreted as indicating total agreement among experts (Escobar & Cuervo, 2008). Construct validity was assessed using exploratory factor analysis (EFA). Factorability was assessed through the Kaiser–Meyer–Olkin (KMO) test and Bartlett's test of sphericity (Polit, 2010).

Confirmatory factor analysis (CFA) was used with a maximum likelihood procedure to test the factorial structure of the scale. Hoyle (1995) and Kline (2016) recommend using at least four adjustment indices. In our study, we have analyzed the following: (a) chi‐square that establishes an acceptable adjustment if the value of *χ* ^2^/*gl* is between 2–5 (Hair et al., 1999); (b) comparative fit index (CFI); (c) Tucker and Lewis's incremental index (TLI); (d) the goodness‐of‐fit index (GFI), where values ≥ 0.90 are deemed adequate (Bentler, 1990; Hair et al., 2010); and (e) the standardized root mean square residual (SRMR), for which values < 0.08 indicate good adjustment of the model (Jöreskog & Sörbom, 1993).

To test the reliability and internal consistency of the questionnaire, the recommendations of Fornell and Larcker (1981) have been followed. Items with a value of Cronbach's *α* < 0.70 were eliminated (Tab achnick et al., 2007). We calculated the item‐total correlation, whose value must be greater than 0.30 (Field, 2013) and the inter‐element correlations were measured with Pearson's correlation coefficients through a dual variation correlation matrix, whose values should be positive and statistically significant (Guyatt et al., 1995). Convergent validity was assessed by correlating the scores of the Spanish versions of the CPS with those of the 10‐item CD‐RISC and CBI using Spearman's *r* with a dual variation correlation matrix. Finally, descriptive statistics (mean, frequencies, standard deviation, skewness and kurtosis) and frequency analysis were applied to analyze each item on the validated scale and the results of the participants.

### 2.4 Participants

Non‐probabilistic sampling (convenience). The sample included 171 family caregivers who were selected from one private hospital and daytime nursing centres in Spain. The inclusion criteria were set as people over 18 years of age, with Spanish nationality, without cognitive impairment, who presented their written consent and excluded health professionals and people taking care of a relative with a moderate, severe or total degree of dependency according to the Barthel Index (Cid & Damián, 1997). In relation to the sample's size, a ratio of 10:1 (subjects to item) was adopted to ensure a sample that was large enough to conduct a factor analysis (Nunnally & Bernstein, 1995). Thus, the minimum number of participants needed to conduct this study was 80. However, the use of a larger sample reduces the sampling error and factor analysis solutions become more stable (MacCallum et al., 1999). Consequently, an attempt was made to get the highest number of participants. The final sample was made up of 171 family caregivers after excluding 1 participant due to anomalous responses.

### 2.5 Instruments

The CPS was developed to assess the preparedness of caregivers for older people who are vulnerable while living at home. It includes eight items (Table 2). The first and second items concern the patient's physical and emotional needs, the next items the caregiver's organizational capacity to provide the caregiving service, to cope with stress while looking after someone, to make caregiving activities enjoyable for both for the patient and the caregiver, to respond to emergencies and to obtain help and information from the health system and the immediate surroundings. The last item considers the caregiver's overall preparation. Each item is scored on a 5‐point Likert‐type scale from 0 (*not at all prepared*)–4 (*very well prepared*). The total score ranges from 0–32, where a high score means better preparedness. A self‐administered pencil‐and‐paper format was chosen. The data were collected through two different instruments: a questionnaire for sociodemographic variables and the Spanish version of the CPS. To evaluate convergent validity, we used the Spanish versions of the 10‐item Connor‐Davidson Resilience Scale (10‐item CD‐RISC; Notario et al., 2011) and Caregiver Burden Interview (CBI; Zarit et al., 1980; Martín et al., 1996).

### 2.6 Ethical considerations

The study was reviewed by and received approval from, district health research ethics committee [2018–37.18] following the recommendations established by the ethical principles for medical research in human beings (World Medical Association Declaration of Helsinki, 2013). All participants were informed about the purpose of the study and the confidentiality of the data collected and signed the informed consent before participating.

## 3 RESULTS

### 3.1 Phase 1: Cross‐culturally adapted scale and sample description

#### 3.1.1 Cross‐culturally adapted scale, usability testing and pre‐testing

Direct translations by both freelance translators were equivalent. The usability test revealed that the caregivers did not understand the wording of item 5 (Archbold et al., 1990). The review committee determined that items 3 and 5 had a similar meaning and included item 7 in the social health system instead of asking only about the health system. Furthermore, they expressed the need to ask about the patient's spiritual needs. Accounting for the fact that religiosity was a category that appeared in the previous qualitative study and that the reviewers considered it, a new item was introduced in the scale. Item 3 was removed from the original scale and spiritual need was introduced. The wording of item 5 was also modified. Comparison with the Italian (Pucciarelli et al., 2014), Swedish (Henriksson et al., 2012) and Turkish (Ugur et al., 2017) versions determined that six of the eight items that made up the scale were the Italian, Swedish and Turkish equivalent of the Spanish words. Regarding the remaining two items, one was equivalent in meaning and the other one was introduced in the Spanish version (spiritual needs). In the reverse translation, the review committee did not detect semantic differences in the remaining items, which ensured the semantic equivalence of the Spanish version. The nurses who participated in the preliminary tests endorsed the understanding of the Spanish version of the scale. The direct translation carried out in the first phase of the adaptation process had to be subjected to modifications after comparison with the other versions of the scale, reverse translation, committee review and pre‐test. The S‐CPS is presented in the appendix.

#### 3.1.2 Sociodemographic description of family caregivers

The sociodemographic data of the population studied are summarized in Table 1. The age of the respondents ranged from less than 45 years old (11%) to over 75 years old (9%), with the most widely represented group having ages between 55–64 years (40%). Most were female (79%) and married (75%). Most had an education level of primary school (33%), secondary school (38%) or university (19%). A considerable part of the sample was employed (37%) or housewives (36%). More than half of the people sampled were daughters in the care of their parents (52%), the income being less than 1,000 euros per month for almost half of the respondents. In 63% of the cases, the caregivers lived with the dependent person. Approximately half of the sample carried out this task for more than 14 hr a day and by duration, 35 of the respondents (20%) had performed it for 2 years, 35% between 3–5 years, 19% between 6–10 years and 25% more than 10 years.

**Table 1:** Family caregivers’ sociodemographic characteristics (*N* = 171)

Characteristics			
	n (%)		n (%)
**Age range**		**Relationship**	
<45 years	19 (11)	Mother/father	30 (17)
45–54 years	44 (26)	Son/daughter	90 (52)
55–64 years	69 (40)	Brother/sister	10 (9)
65–75 years	24 (14)	Husband/wife	28 (16)
>75 years	16 (9)	Daughter in law/son in law	8 (5)
**Gender**		Others	6 (3)
Male	36 (21)	**Rent**	
Female	136 (79)	<501€	14 (8)
**Marital status**		501**–**1000€	65 (38)
Married	129 (75)	>1,000€	91 (53)
Single	26 (15)	**Living together**	
Widower	9 (5)	Yes	109 (63)
Divorced	4 (2)	No	63 (37)
In couple	4 (2)	**Hours/day caring**	
**Education level**		2**–**8 hr/day	52 (30)
No studies	17 (10)	9–13 hr/day	42 (24)
Primary studies	56 (33)	≥14 hr/day	78 (45)
Secondary studies	66 (38)	**Years caring**	
University studies	33 (19)	0–2 years	35 (20)
**Employment situation**		3–5 years	61 (35)
Employee	64 (37)	6–10 years	33 (19)
Retired	26 (15)	>10 years	43 (25)
Unemployed	20 (12)		
Housewife	62 (36)		

### 3.2 Phase 2: psychometric evaluation

#### 3.2.1 Content validity

Content validity was addressed through the opinion of eight experts. Their results were evaluated using the Kendall W coefficient, whose value for the scale was 1,000 (*p* ≤ .000). There were no statistically significant differences between the experts’ variables, and the results were compatible with each other. As a result, the need to include spiritual needs of the caregiver was indicated as another domain in the caregiver's role, included in the questionnaire as Item 3.

#### 3.2.2 Construct validity

**Figure 1:**
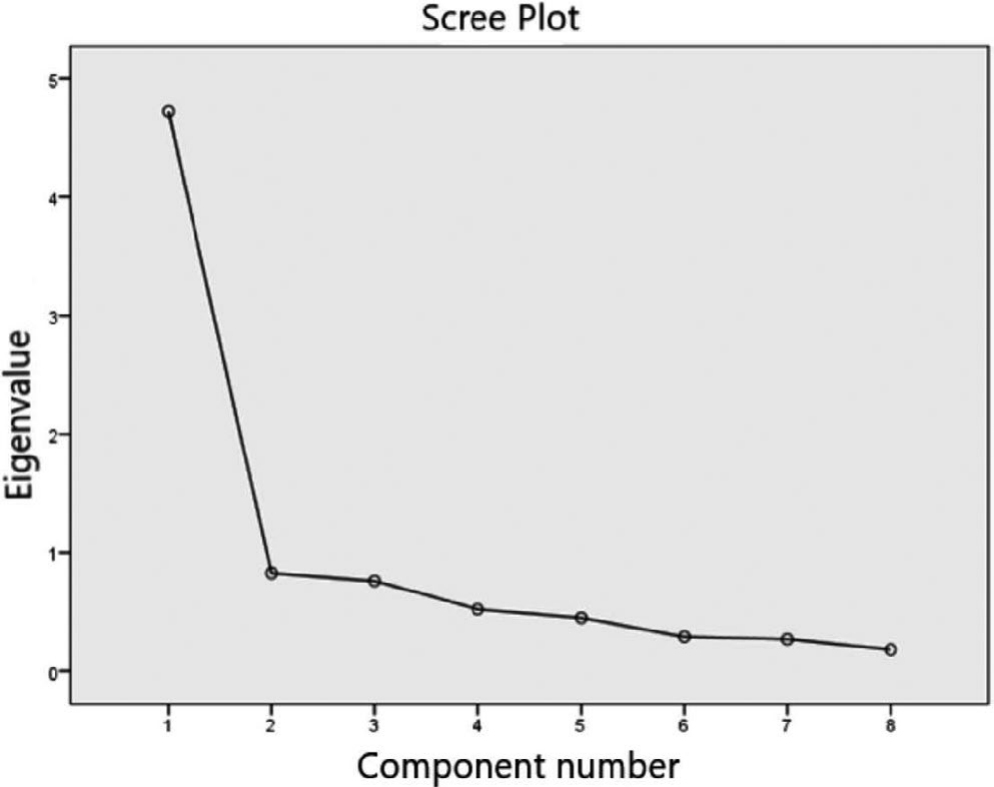
Scree Plot

Construct validity was assessed using the EFA. The significance of Bartlett's test of sphericity (∑^2^ = 780.790; *df* = 28; *p* < .001) and the size of the KMO measure of sampling adequacy (KMO = 0.88) revealed an adequate variance in the items of S‐CPS to perform the EFA (Tabachnick & Fidell, 2001). The average for all the extracted commonalities was 0.59 (4.725/8). The scree plot revealed a one‐factor solution (Figure 1). The Spanish version of the scale explains 59% of the total variance, higher than the 50% level recommended by Fornell and Larcker (1981). EFA showed that factor loads ranged between 0.577–0.902 (Table 2). These values demonstrated that the sample size was sufficient to perform factor analysis.

**Table 2:** Internal consistency, reliability and principal components factor loadings of the S‐CPS in family caregivers (*N* = 171)

Item	I R I	*r*_jx_	*α* − *X*	Loadings
1. How prepared do you feel to meet your family member's physical needs?	0.64	0.712	0.877	0.806
2. How prepared do you feel to take care of your family member's emotional needs?	0.64	0.776	0.871	0.837
3. How prepared do you feel to meet your family member's spiritual needs?	0.36	0.484	0.902	0.577
4. How prepared do you feel to face the stress of caring for someone else?	0.59	0.707	0.877	0.796
5. How prepared do you feel to carry out care in a way that is pleasant for you?	0.61	0.705	0.877	0.790
6. How prepared do you feel to respond and manage emergencies that may arise for your family member?	0.45	0.624	0.885	0.711
7. How prepared do you feel to obtain the necessary information and help that your family member needs from the social health system?	0.41	0.587	0.888	0.682
8. In general, how prepared do you feel to take care of your family member?	0.77	0.845	0.865	0.902

Abbreviations: IRI, item reliability index; *r_jx_*, item‐total correlation; *α − X*, Cronbach's alpha without the item.

**Figure 2:**
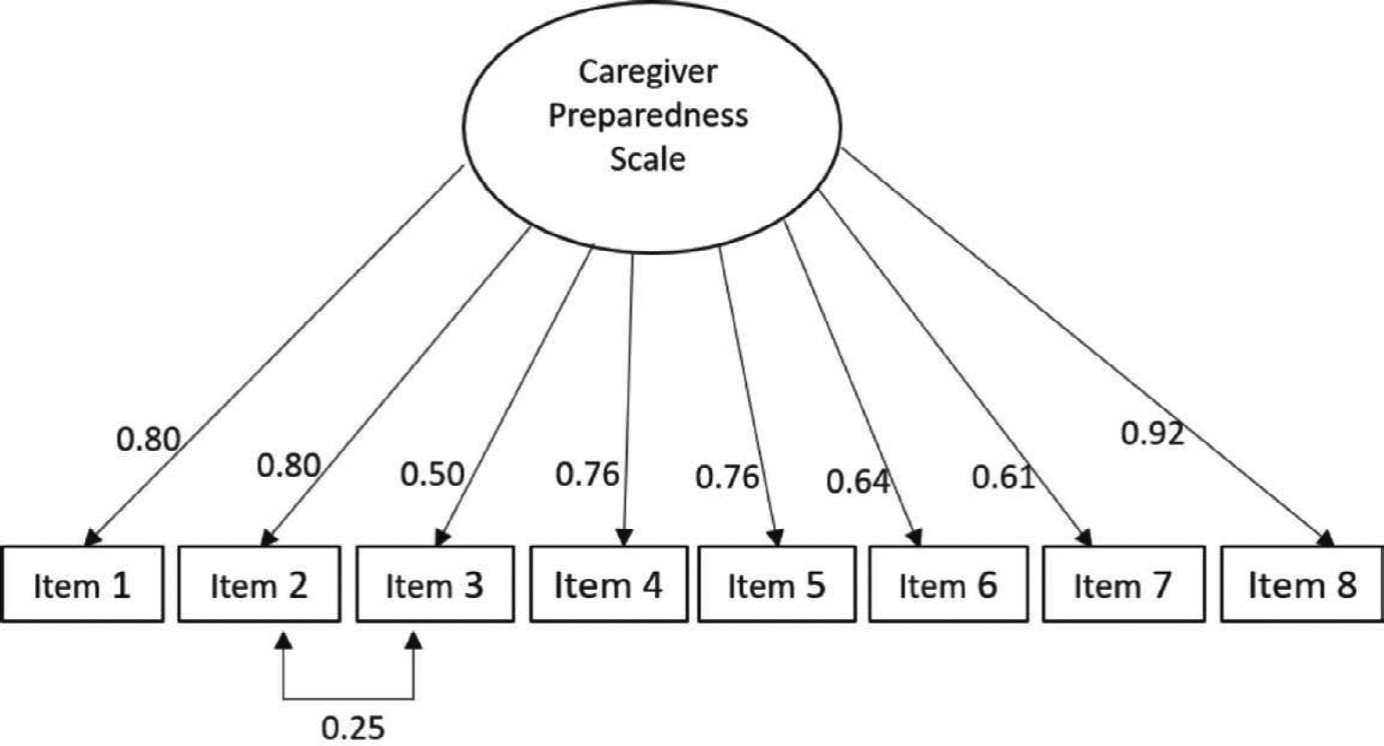
Confirmatory factor analysis of the Spanish version CPS in family caregivers

The CFA confirmed the existence of a single factor (Figure 2). The model showed appropriate results for the fitness indices except chi‐square (*∑* ^2^ (20, *N* = 171) = 80.2, *p* < .001; CFI = 0.92; TLI = 0.90; GFI = 0.90; SRMR = 0.059). A more detailed analysis of the modification error rates found items 2 and 3 to be closely related. The load factors for CPS ranged from 0.50–0.92 and were statistically significant.

#### 3.2.3 Reliability analysis

The results show that all the items contribute to the scale (Table 2). The coefficients of the item reliability index and the total item correlation (*r*_jx_) were higher than the recommended level 0.30 (Field, 2013). Cronbach's *α* was 0.89. Removing any item from the scale did not improve the reliability of the scale. Inclusion of Item 3 yielded a slight non‐significant increase in Cronbach's alpha. These results are considered as indicating good reliability (Hair et al., 1999).

#### 3.2.4 Convergent validity

The correlations between the scores of the S‐CPS, 10‐item CD‐RISC and CBI show the S‐CPS to present convergent validity (Table 3). Correlations were in the expected directions and were statistically significant (*p* < .01), ranging from − 0.285 (between S‐CPS and CBI) –0.525 (between S‐CPS and 10‐item CD‐RISC). Therefore, a high S‐CPS score was associated with low levels of burden and high levels of caregiver resilience.

**Table 3:** Convergent validity of the Spanish version of the CPS in family caregivers (*N* = 171)

	S‐CPS	10‐item CD‐RISC	CBI
S‐CPS	1.00		
10‐item CD‐RISC	0.525**	1.00	
CBI	−0.285**	−0.268**	1.00

Abbreviations: 10‐item CD‐RISC, Connor‐Davidson Resilience Scale; CBI, Caregiver Burden Interview; S‐CPS, Spanish Caregiver Preparedness Scale.

** *p* < .01.

#### 3.2.5 Item analysis

The positive correlations between the items show that all of them contribute to the scale (Table 4). All items are necessary to measure the construct to be measured. Pearson's correlation coefficients varied between 0.305–0.765 and were statistically significant (*p* < .01). All items are within accepted values (Guyatt et al., 1995).

**Table 4:** Correlation matrix inter‐elements of the Spanish version of the CPS in family caregivers (*N* = 171)

	Item 1	Item 2	Item 3	Item 4	Item 5	Item 6	Item 7	Item 8
Item 1	1.00							
Item 2	0.642**	1.00						
Item 3	0.305**	0.575**	1.00					
Item 4	0.557**	0.589**	0.336**	1.00				
Item 5	0.539**	0.616**	0.395**	0.705**	1.00			
Item 6	0.567**	0.485**	0.344**	0.479**	0.412**	1.00		
Item 7	0.476**	0.468**	0.315**	0.453**	0.401**	0.547**	1.00	
Item 8	0.765**	0.717**	0.424**	0.691**	0.706**	0.564**	0.571**	1.00

** *p* < .01.

### 3.3 Phase 3: Descriptive statistics of the S‐CPS items

Tables 5 and 6 present the descriptive analysis and frequency analysis of a scale with eight items validated for the Spanish setting. The descriptive statistics of the scale shows an average degree of caregiver preparedness, with the mean item score ranging between 2.16 (range 0–4) for Item 4–2.79 for Item 6. All items were within the standard criteria of asymmetry and kurtosis (Groeneveld & Meeden, 1984).

**Table 5:** Descriptive statistics of the Spanish version CPS items in family caregivers (*N* = 171)

	Mean	*SD*	Skewness	Kurtosis
Item 1	2.66	1.04	−0.55	−0.41
Item 2	2.41	1.01	−0.69	0.47
Item 3	2.29	1.20	−0.57	−0.66
Item 4	2.16	1.09	−0.46	−0.50
Item 5	2.54	1.00	−0.73	0.38
Item 6	2.79	0.93	−1.01	1.07
Item 7	2.70	0.99	−0.90	0.62
Item 8	2.64	0.94	−0.90	0.93

Abbreviation: *SD*, standard deviation.

**Table 6:** Frequency analysis of the Spanish version of the CPS items in family caregivers (*N* = 171)

	Punctuation item, %^a^				
	0	1	2	3	4
Item 1	2.3	14.0	20.3	41.3	21.8
Item 2	5.8	11.6	27.3	45.3	9.3
Item 3	11.6	14.0	19.8	42.4	11.6
Item 4	9.9	15.1	30.8	36.6	7.0
Item 5	5.2	7.6	28.5	44.2	14.0
Item 6	2.9	7.6	15.7	54.7	18.6
Item 7	4.1	8.1	19.2	50.0	18.0
Item 8	4.1	6.4	24.4	51.2	13.4

^a^ Answer options: 0 = not at all prepared, 1 = not very well prepared, 2 = minimally prepared, 3 = well prepared; 4 = very well prepared.

The scores on the Likert scale range from 0 points (*not prepared at all*)–4 points (*very well prepared*) and the average of the total scale score in our sample was 20.19 (*SD* 6.2) indicating that 53.8% were above the cut‐off mark. The minimum and maximum scores obtained were 0 and 32 points, respectively. The highest percentage of the frequency distribution fell around a score of 2 (*minimally prepared*) and 3 (*well prepared*). Considering caregivers who answered 0 (*not at all prepared*) on some of the items, Item 3 showed the highest percentage of responses of 0. Higher percentages of the caregivers who answered 4 (*very well prepared*) on some of the items did so on Item 1.

## 4 DISCUSSION

The profile of the caregiver in most recent studies is a woman (>55%), married (>60%), with a level of education of primary school, unemployed, with limited economic opportunities, usually living with the person she is looking after and who, in addition to providing caregiving to the dependent person, also performs household tasks and takes care of a family (Casado & Ruíz, 2016; Delicado et al., 2004; Petruzzo et al., 2017; Pucciarelli et al., 2014). The results of our study corroborate those of earlier studies. In 79% of cases, the caregiver is a middle‐aged woman, married (75%), either unemployed or working as a housewife (48%) and, in 63% of the cases, lives with the person needing daily care and thus must combine her own family life with caregiving. However, our sample has a significant number of subjects with secondary (38%) and university education (19%) and more than half of the sample (53%) has an income higher than 1,000 euros. This level of economic opportunity, contrary to previous studies (Ruíz‐Adame et al., 2017), supports the idea that the socioeconomic profile of the caregiver could be changing in recent years as caregivers remain employed in addition to performing their caregiving role (Alpass et al., 2017).

The judgment of the panel of experts determined the need to address spirituality. We therefore included this aspect of caregiving, which had been unexplored in previous versions. Spirituality is important for the well‐being of the dependent person (Özdemir et al., 2020), and the religious dimension can positively affect the ability to cope with traumatic experiences (Stratta et al., 2013) and transform the act of caregiving into a rewarding experience (Lalani et al., 2018). In this same line of thought, “low spirituality” is considered a risk factor for low resilience (Min et al., 2013), whereas adequate spiritual caregiving improves the quality of life and positively influences coping with a disease in both the patient and close relatives (Bermejo et al., 2013). On the other hand, in our AFC study, we found a covariance between two items of the scale that could be related to the readiness to meet emotional and spiritual needs. Regarding these items, in our sample of caregivers, one out of four caregivers affirmed that they are not at all prepared or not very well prepared to attend to the spiritual needs of their family and may not be meeting these needs. Taking this into account with the results of our study, we posit that caregivers should not only try to identify those needing emotional attention, but should be trained in these areas to attend to spiritual needs if they are demanded by those who they look after.

According to the EFA, only one factor has been obtained (Figure 1), in the same way as with the original scale (Archbold et al., 1990) and other versions (Petruzzo et al., 2017; Pucciarelli et al., 2014; Ugur et al., 2017). The scale explains 59% of the total variance, whereas in previous studies 57.3% (Petruzzo et al., 2017), 65% (Pucciarelli et al., 2014) and 56% (Ugur et al., 2017) of the total variance was explained. All results were above the recommended level of 50% (Fornell & Larcker, 1981; Sencan, 2005). The CFA confirmed the one‐dimensionality of the scale found in previous validations (Petruzzo et al., 2017; Pucciarelli et al., 2014; Ugur et al., 2017). The loads for each item were between 0.50–0.92, showing a high impact of the items on the scale (Figure 2). Item loads in previous studies ranged from 0.51–0.84 (Ugur et al., 2017) and from 0.74–0.89 (Pucciarelli et al., 2014). Correlations between elements showed values between 0.305–0.765, falling within the recommended range between 0.15–0.85 (Clark & Watson, 1995). Thus, we can affirm that all items measure characteristics of the same construct.

We evaluated the internal consistency of the scale using Cronbach's *α*, obtaining a value of *α* = 0.89 for the Spanish version of the CPS with eight items. Values above 0.70 are considered as indicating acceptable reliability (Streiner, 2003). Previous CPS validations also demonstrated high Cronbach's *α*. In a sample of patients with heart failure and stroke survivors, *α* = 0.91 and 0.94 were obtained, respectively (Petruzzo et al., 2017; Pucciarelli et al., 2014), while in cancer patients or terminally ill patients, reliability indices of 0.88, 0.92 and 0.90 were obtained, respectively (Henriksson et al., 2012; Hudson and Hayman‐White, 2006; Ugur et al., 2017). In our study, the item's total correlation coefficients ranged from 0.48–0.85, with the recommended values being above 0.30 (Field, 2013), while a value of 0.63 was found in previous studies (Petruzzo et al., 2017; Pucciarelli et al., 2014). When eliminating the item, Cronbach's *α* decreased (*α* ≤ 0.89), showing that all the items are important and contribute to the scale, with Item 3 showing the lowest values. This coefficient has been previously corroborated in other validations (Petruzzo et al., 2017; Pucciarelli et al., 2014).

The convergent validity test of the S‐CPS yielded adequate results. We hypothesized that if the S‐CPS measures readiness, caregivers who scored high on the Spanish version of the CPS would score high on the 10‐item CD‐RISC and low on the CBI. Our results support this hypothesis. Only two research groups have tested the convergent validity of the CPS (Henriksson et al., 2012; Petruzzo et al., 2017). These results indicate that the S‐CPS can be correlated with other aspects such as resilience or work overload that caregivers may experience and that in turn could define different types of caregivers.

The descriptive analysis of the items shows an average response score of 2.52 on the 5‐point Likert scale (range 0–4). This result indicates that our sample has a level of preparation between 2 (*minimally prepared*)–3 (*well prepared*). In previous studies, lower values were obtained (2.11 and 1.93), which may indicate a lower level of preparedness (Petruzzo et al., 2017; Pucciarelli et al., 2014). Regarding the frequency analysis, it should be noted that one out of every four of the caregivers acknowledged not being prepared at all or not very well prepared to face the stress involved in caregiving and only 7% stated that they were highly prepared to manage it. Caregiver stress is related to caregiving capacity, which decreases when stress increases (Coppetti et al., 2019). We can affirm that many caregivers feel stress from caregiving, especially those who dedicate many hours per day to caregiving or who have done it for many years (based on our own unpublished research results). This could be related to the fact that caregivers experience mental‐health‐related problems such as depression and anxiety disorders more frequently than the general population (Strada, 2019; Hernández et al., 2019). As mentioned above, caregivers express that they are not able to properly manage emotional or spiritual needs. However, 63% of the respondents felt prepared or very well prepared to face the physical needs of those they looked after and more than 70% felt capable of managing emergencies. We may thus generalize to infer that caregivers approach the objective aspects of caregiving better than the subjective aspects. Nevertheless, accounting for the fact that most caregivers do not have previous training in caregiving, expanding their knowledge of both the technical capacity to address physical needs and the management of stress and frustration is strongly recommended.

The S‐CPS presents adequate psychometric properties and provides healthcare personnel with a valid and reliable tool to measure the caregiver's preparedness while attending dependent people. Caregiving must always be adapted to meet the diverse needs of each pathology. The scale is also applied to obtain correlations with other aspects related to caregiving (Henriksson & Årestedt, 2013; Petruzzo et al., 2019; Schumacher et al., 2007; Vellone et al., 2020). CPS has been used to correlate levels of caregiver preparedness and depression, establishing that preparedness reduces depression in caregivers who handle patients with heart problems (Petruzzo et al., 2019). This same group of researchers recently reported that the level of preparedness influences the management and maintenance of the caregiver's own needs (Vellone et al., 2020). Other researchers have found that respondents with more training showed higher levels of hope and reward and lower levels of anxiety (Henriksson & Årestedt, 2013). The creators of the CPS determined that caregivers of cancer patients were at risk of mood disturbances when their preparedness was low (Schumacher et al., 2007). As other authors have shown, caregiver training comes in many forms from nurses who offer basic knowledge for the management of complex pathologies that include patients with neoplasm (Giarelli & Ed, 2003). However, the CPS has also been applied to determine the level of knowledge of caregivers of patients undergoing rehabilitation for other diverse pathologies (Stone, 2014). Our study includes caregivers of patients with very heterogeneous pathologies. Although this may be a limitation, the fact that all caregiving recipients have low Barthel indices (heavy dependence) indicates that they all needed caregiving most of the day. Furthermore, our adaptation of the CPS demonstrated validity and reliability, indicating that the scale may be useful for the general caregiver population regardless of the type of caregiving. However, it would be interesting to correlate the values of the readiness to apply caregiving with other characteristics of the caregiver role.

In the adapted version of the CPS, all the validity factor tests were positive and all the reliability indices (Cronbach's *α*, etc.) were above the threshold values. The results of this study demonstrate that the S‐CPS has strong psychometric support as measuring the preparation of the family caregiver. Therefore, health personnel could use it in clinical practice to identify the level of preparedness of the family caregiver and, in the case of caregivers with a low level of preparation, establish specific interventions.

### 4.1 Limitations

One limitation of this study is that a sample from only one geographical region was used. Despite this, on the basis of a population study carried out in southern Spain, we believe that the sample is representative of the whole country. Further testing with more varied and representative samples should, therefore, be undertaken to ratify the present findings and improve the scale. Another limitation of the study is that neither the sensitivity of the scale to measure changes, nor the test–retest reliability has been analysed. Both tests should be evaluated formally in further studies to support its usefulness as in the Italian version of the scale, which offers the test–retest (Pucciarelli et al., 2014).

## 5 CONCLUSION

The Spanish version of the CPS shows validity and reliability. All the items on the scale contribute significantly to the scale. Low values on the scale indicate that the caregivers reported poor preparedness for caregiving and attending to the emotional or physical needs of dependent people living at home. The Spanish version of the CPS is thus a useful tool to measure the level of preparedness of caregivers in the Spanish setting.

## CONFLICTS OF INTEREST

The authors declare no conflicts of interest.

## AUTHOR CONTRIBUTIONS

CRG performed substantial conception and experimental design. BGB carried out the data collection. CRG and BGB performed the analysis and interpretation of the data. BGB drafted the paper and CRG critically revised it. Both authors are responsible for all aspects of the work and approve the final version for publication.

## Data Availability

The data generated for this study are available on request to the corresponding author.

## Appendix 1

### Escala de preparación para el cuidado

Sabemos que las personas pueden sentirse bien preparadas en algunos aspectos para cuidar de otra persona y no tan bien preparadas para hacerlo en otras facetas. Nos gustaría saber cómo de preparado se siente para cada una de las siguientes cuestiones., incluso si no está realizando un cuidado de este tipo ahora mismo.

**Table:**

A continuación, le vamos a pedir que valore como de preparado se siente para:	Nada preparado	No muy bien preparado	Mínimamente preparado	Bien preparado	Muy bien preparado
1. Atender las necesidades físicas de su familiar.	0	1	2	3	4
2. Cuidar de las necesidades emocionales de su familiar.	0	1	2	3	4
3. Atender las necesidades espirituales de su familiar.	0	1	2	3	4
4. Afrontar el estrés que implica el cuidado de otra persona.	0	1	2	3	4
5. Llevar a cabo los cuidados de forma agradable para usted.	0	1	2	3	4
6. Responder y gestionar las emergencias que le puedan surgir a su familiar.	0	1	2	3	4
7. Conseguir la información y ayuda necesaria que su familiar necesita desde el sistema sociosanitario.	0	1	2	3	4
8. En general, ¿cómo de preparado se siente para cuidar a su familiar?	0	1	2	3	4

## References

[nop2732-bib-0001] Abellán, A. , Esparza, C. , & Pérez, J. (2011). Evolución y estructura de la población en situación de dependencia. Cuadernos De Relaciones Laborales, 29, 43–67. 10.5209/rev_CRLA.2011.v29.n1.2

[nop2732-bib-0002] Alpass, F. , Keeling, S. , Allen, J. , Stevenson, B. , & Stephens, C. (2017). Reconciling work and caregiving responsibilities among older workers in New Zeland. Journal of Cross‐Cultural Gerontology, 32(3), 323–337. 10.1007/s10823-017-9327-3 28664423

[nop2732-bib-0003] Archbold, P. G. , Stewart, B. J. , Greenlick, M. R. , & Harvath, T. (1990). Mutuality and preparedness as predictors of caregiver role strain. Research in Nursing & Health, 13(6), 375–384. 10.1002/nur.4770130605 2270302

[nop2732-bib-0004] Beaton, D. E. , Bombardier, C. , Guillemin, F. , & Ferraz, M. B. (2000). Guidelines for the process of cross‐cultural adaptation of self‐report measures. Spine, 25(24), 3186–3191. 11124735 10.1097/00007632-200012150-00014

[nop2732-bib-0005] Bentler, P. M. (1990). Comparative fit indexes in structural models. Psychological Bulletin, 107(2), 238–246. 10.1037/1082-989X.3.4.424 2320703

[nop2732-bib-0006] Bermejo, J. C. , Lozano, B. , Villacieros, M. , & Gil, M. (2013). Medicina paliativa de los usuarios: Valoración y vivencia de los usuarios. Medicina Paliativa, 20(3), 93–102. 10.1016/j.medipa.2012.05.004

[nop2732-bib-0007] Bódalo, E. (2010). Cambios en los estilos de vida de las cuidadoras de personas dependientes. Portularia, 10(1), 85–97. 10.5218/prts.2010.0007

[nop2732-bib-0008] Carter, J. H. , Lyons, K. S. , Stewart, B. J. , Archbold, P. G. , & Scobee, R. (2010). Does age make a difference in caregiver strain? Comparison of young versus older caregivers in early‐stage Parkinson’s disease. Movement Disorders, 25(6), 724–730. 10.1002/mds.22888 20201024

[nop2732-bib-0009] Cid, J. , & Damián, J. (1997). Valoración de la discapacidad física: El índice de Barthel. Revista Española De Salud Pública, 71(2), 127–137. 9546856

[nop2732-bib-0010] Clark, L. A. , & Watson, D. (1995). Constructing validity: Basic issues in objective scale development. American Psychological Association, 7(3), 309–319. 10.1037/1040-3590.7.3.309

[nop2732-bib-0011] Coppetti, L. D. C. , Girardon‐Perlini, N. M. O. , Andolhe, R. , Silva, L. M. C. D. , Dapper, S. N. , & Noro, E. (2019). Caring ability, burden, stress and coping of family caregivers of people in cancer treatment. Revista Brasileira De Enfermagem, 72(6), 1541–1546. 10.1590/0034-7167-2018-0605 31644742

[nop2732-bib-0012] Crespo, M. , Fernández‐Lansac, V. , & Soberón, C. (2014). Spanish version of the CD‐RISC resilience scale for chronic stress situations. Behavioral Psychology, 22, 219–238.

[nop2732-bib-0013] Escobar, J. , & Cuervo, A. (2008). Validez de contenido y juicio de expertos: una aproximación a su utilización. Avances en Medición, 6, 27–36. 10.1016/0032-3861(78)90049-6

[nop2732-bib-0014] Escobar‐Bravo, M. A. (2004). Adaptación transcultural de instrumentos de medida relacionados con la salud (Cross‐cultural adaptation of health‐related measuring instruments). Enfermería Clínica, 14(2), 102–106. 10.1016/S1130-8621(04)73863-2

[nop2732-bib-0015] Field, A. (2013). Discovering statistics using IBM SPSS statistics. SAGE.

[nop2732-bib-0016] Fornell, C. , & Larcker, D. F. (1981). Evaluating structural equation models with unobservable variables and measurement. Marketing Reserarch, 18(1), 39–51. 10.1177/002224378101800104

[nop2732-bib-0017] Fortin, M. , Soubhi, H. , Hudon, C. , Bayliss, E. A. , & van den Akker, M. (2007). Multimorbidity's many challenges. BMJ, 334(7602), 1016–1017. 17510108 10.1136/bmj.39201.463819.2C PMC1871747

[nop2732-bib-0018] Garratt, A. M. , Ruta, D. A. , Abdalla, M. I. , Buckingham, J. K. , & Russell, I. T. (1993). The SF 36 health survey questionnaire: An outcome measure suitable for routine use within the NHS? British Medical Journal, 306, 1440–1444. 10.1136/bmj.306.6890.1440 8518640 PMC1677883

[nop2732-bib-0019] Giarelli, E. , & Ed, D. (2003). Caring for a spouse after prostate surgery: The preparedness needs of wives. Journal of Family Nursing, 9(4), 453–485.

[nop2732-bib-0020] Groeneveld, R. A. , & Meeden, G. (1984). Measuring skewness and kurtosis. Journal of the Royal Statistical Society: Series D (The Statistician), 33(4), 391–399.

[nop2732-bib-0021] Guillemin, F. , Bombardier, C. , & Beaton, D. (1993). Cross‐cultural adaptation of health‐related quality of life measures: Literature review and proposed guidelines. Journal of Clinical Epidemiology, 46(12), 1417–1432. 10.1016/0895-4356(93)90142-N 8263569

[nop2732-bib-0022] Guillemin, F. , Brianqon, S. , & Pourel, J. (1992). Validity and discriminant ability of a French version of the Health Assessment Questionnaire in early RA. Disability and Rehabilitation, 14, 71–77. 1600184 10.3109/09638289209167073

[nop2732-bib-0023] Gutiérrez, B. , & Romero‐Grimaldi, C. (2018). En la Salud y en la Enfermedad: El relato de una cuidadora. Archivos de la Memoria, 15. Retrieved from http://ciberindex.com/c/am/e01516

[nop2732-bib-0024] Guyatt, G. , Walter, S. , Shannon, H. , Cook, D. , Jaeschke, R. , & Heddle, N. (1995). Basic statistics for clinicians: 4. Correlation and regression. Canadian Medical Association Journal, 152(4), 497–504. 7859197 PMC1337703

[nop2732-bib-0025] Hair, J. F. , Anderson, R. E. , Tatham, R. L. , & Black, W. C. (1999). Análisis multivariante. Prentice Hall.

[nop2732-bib-0026] Hair, J. F. , Black, W. C. , Babin, B. J. , & Anderson, R. E. (2010). Multivariate data analysis. Prentice Hall.

[nop2732-bib-0027] Hendricson, W. D. , Jon Russell, I. , Prihoda, T. J. , Jacobson, J. M. , Rogan, A. , Bishop, G. D. , & Castillo, R. (1989). Development and initial validation of a dual language English‐Spanish format for the Arthritis Impact Measurement Scales. Arthritis and Rheumatism, 32, 1153–1159. 10.1002/anr.1780320915 2775323

[nop2732-bib-0028] Henriksson, A. , Andershed, B. , Benzein, E. , & Årestedt, K. (2012). Adaptation and psychometric evaluation of the Preparedness for Caregiving Scale, Caregiver Competence Scale and Rewards of Caregiving Scale in a sample of Swedish family members of patients with life‐threatening illness. Palliative Medicine, 26(7), 930–939. 10.1177/0269216311419987 21908520

[nop2732-bib-0029] Henriksson, A. , & Årestedt, K. (2013). Exploring factors and caregiver outcomes associated with feelings of preparedness for caregiving in family caregivers in palliative care: A correlational, cross‐sectional study. Palliative Medicine, 27(7), 639–643. 10.1177/0269216313486954 23670720

[nop2732-bib-0030] Henriksson, A. , Hudson, P. , Öhlen, J. , Thomas, K. , Holm, M. , Carlander, I. , Hagell, P. , & Årestedt, K. (2015). Use of the Preparedness for Caregiving Scale in palliative care: A Rasch evaluation study. Journal of Pain and Symptom Management, 50(4), 533–541. 10.1016/j.jpainsymman.2015.04.012 26004399

[nop2732-bib-0031] Hernández, M. A. , Fernández, M. J. , Blanco, M. A. , Alves, M. T. , Fernández, M. J. , Souto, A. I. , & Clavería Fontán, A. (2019). Depresión y sobrecarga en el cuidado de personas mayores. Revista Española De Salud Publica, 93, 1–10.31378780

[nop2732-bib-0032] Hoyle, R. H. (1995). Structural equation modeling: Concepts, issues and applications. SAGE.

[nop2732-bib-0033] Hudson, P. L. , & Hayman‐White, K. (2006). Measuring the psychosocial characteristics of family caregivers of palliative care patients: Psychometric properties of nine self‐report instruments. Journal of Pain and Symptom Management, 31(3), 215–228. 10.1016/j.jpainsymman.2005.07.010 16563316

[nop2732-bib-0034] Jöreskog, K. G. , & Sörbom, D. (1993). LISREL 8: Structural equation modeling with the SIMPLIS command language. Scientific Software International.

[nop2732-bib-0035] Kline, R. B. (2016). Principles and practice of structural equation modeling. The Guilford Press.

[nop2732-bib-0036] Kneeshaw, M. F. , Considine, R. M. , & Jennings, J. (1999). Mutuality and preparedness of family caregivers for elderly women after bypass surgery. Applied Nursing Research, 12(3), 128–135. 10.1016/S0897-1897(99)80034-2 10457623

[nop2732-bib-0037] Lalani, N. , Duggleby, W. , & Olson, J. (2018). Spirituality among family caregivers in palliative care: An integrative literature review. International Journal of Palliative Nursing, 24(2), 80–91. 10.12968/ijpn.2018.24.2.80 29469645

[nop2732-bib-0038] López, M. J. , Orueta, R. , Gómez‐Caro, S. , Sánchez, A. , Carmona, J. , & Alonso, F. J. (2009). El rol de Cuidador de personas dependientes y sus repercusiones sobre su Calidad de Vida y su Salud. Revista Clínica de Medicina de Familia, 2(7), 332–334. 10.4321/S1699-695X2009000200004

[nop2732-bib-0039] MacCallum, R. C. , Widaman, K. F. , Zhang, S. , & Hong, S. (1999). Sample size in factor analysis. Psychological Methods, 4, 84–99. 10.1037/1082-989X.4.1.84

[nop2732-bib-0040] Martín, M. , Salvadó, I. , Nadal, S. , Miji, L. C. , Rico, J. M. , Lanz, P. , & Taussing, M. I. (1996). Adaptación para nuestro medio de la escala de sobrecarga del cuidador (Caregiver Burden Interview) de Zarit. Revista Multidisciplinar De Gerontología, 6(4), 338–346.

[nop2732-bib-0041] Min, J.‐A. , Jung, Y.‐E. , Kim, D.‐J. , Yim, H.‐W. , Kim, J.‐J. , Kim, T.‐S. , Lee, C.‐U. , Lee, C. , & Chae, J.‐H. (2013). Characteristics associated with low resilience in patients with depression and/or anxiety disorders. Quality of Life Research, 22(2), 231–241. 10.1007/s11136-012-0153-3 22485024

[nop2732-bib-0042] Notario, B. , Solera, M. , Serrano, M. D. , Bartolomé, R. , García, J. , & Martínez, V. (2011). Reliability and validity of the Spanish version of the 10‐item Connor‐Davidson Resilience Scale (10‐item CD‐RISC) in young adults. Health and Quality of Life Outcomes, 9, 63. 10.1186/1477-7525-9-63 21819555 PMC3173284

[nop2732-bib-0043] Nunnally, J. C. , & Bernstein, I. H. (1995). Análisis Factorial I: El modelo general y la condensación de la varianza (Factor Analysis I: The General Model and Variance Condensation). In J. C. Nunnally , & I. H. Bernstein (Eds.), Teoría Psicométrica (Psychometric Theory), 3rd ed. (pp. 505–526). McGraw‐Hill.

[nop2732-bib-0044] World Health Organization (2015). World report on ageing and health. https://apps.who.int/iris/handle/10665/186463

[nop2732-bib-0045] Özdemir, F. , Doğan, S. , & Atayoğlu, A. T. (2020). Psychosocial problems of family caregivers of palliative care patients and their spiritual coping styles. Perspectives in Psychiatric Care, 56(2), 1–6. 10.1111/ppc.12479 32017126

[nop2732-bib-0046] Petruzzo, A. , Biagioli, V. , Durante, A. , Emberti, L. , Agostino, F. D. , Alvaro, R. , & Vellone, E. (2019). Patient education and counseling influence of preparedness on anxiety, depression and quality of life in caregivers of heart failure patients: Testing a model of path analysis. Patient Education and Counseling, 102(5), 1021–1028. 10.1016/j.pec.2018.12.027 30611564

[nop2732-bib-0047] Petruzzo, A. , Paturzo, M. , Buck, H. G. , Barbaranelli, C. , D'Agostino, F. , Ausili, D. , Alvaro, R. , & Vellone, E. (2017). Psychometric evaluation of the Caregiver Preparedness Scale in caregivers of adults with heart failure. Research in Nursing & Health, 40(5), 470–478. 28884832 10.1002/nur.21811

[nop2732-bib-0048] Polit, D. F. (2010). Statistics and data analysis for nursing research, 2nd ed. Pearson.

[nop2732-bib-0049] Pucciarelli, G. , Savini, S. , Byun, E. , Simeone, S. , Barbaranelli, C. , Vela, R. J. , & Vellone, E. (2014). Psychometric properties of the Caregiver Preparedness Scale in caregivers of stroke survivors. Heart and Lung, 43(6), 555–560. 10.1016/j.hrtlng.2014.08.004 25239706

[nop2732-bib-0050] Roguero‐García, J. (2009). Distribución en España del cuidado formal e informal a las personas de 65 y más años en situación de dependencia. Revista Española Salud Pública, 83(3), 393–405. 10.1590/S1135-57272009000300005 19701571

[nop2732-bib-0051] Schumacher, K. L. , Stewart, B. J. , & Archbold, P. G. (2007). Mutuality and preparedness moderate the effects of caregiving demand on cancer family caregiver outcomes. Nursing Research, 56(6), 425–433. 10.1097/01.NNR.0000299852.75300.03 18004189

[nop2732-bib-0052] Sencan, H. (2005). Reliability and Validity in Social and Behavioral Measures. Ankara. A Work Book (in Turkish).

[nop2732-bib-0053] Stone, K. (2014). Enhancing preparedness and satisfaction of caregivers of patients discharged from an inpatient rehabilitation facility using an interactive website. Rehabilitation Nursing, 39(2), 76–85. 10.1002/rnj.123 24038541

[nop2732-bib-0054] Strada, E. A. (2019). Psychosocial issues and bereavement. Primary Care: Clinics in Office Practice, 46(3), 373–386. 10.1016/j.pop.2019.05.004 31375187

[nop2732-bib-0055] Stratta, P. , Capanna, C. , Riccardi, I. , Perugi, G. , Toni, C. , Dell’Osso, L. , & Rossi, A. (2013). Spirituality and religiosity in the aftermath of a natural catastrophe in Italy. Journal of Religion and Health, 52(3), 1029–1037. 10.1007/s10943-012-9591-z 22395757

[nop2732-bib-0056] Streiner, D. L. (2003). Starting at the beginning: An introduction to coefficient alpha and internal consistency. Personality Assessment, 80(1), 99–103. 10.1207/S15327752JPA8001_18 12584072

[nop2732-bib-0057] Tabachnick, B. G. , & Fidell, L. S. (2001). Using Multivariate Statistics, 4th ed. Allyn and Bacon.

[nop2732-bib-0058] Tabachnick, B. G. , Fidell, L. S. , & Ullman, J. B. (2007). Using multivariate statistics. Pearson.

[nop2732-bib-0059] Ugur, O. , Elçigil, A. , Aslan, D. , & Paçal, S. (2017). The psychometric properties of the Preparedness Scale of the family Care Inventory: The turkish version. International Journal of Caring Sciences, 10(2), 657–668.

[nop2732-bib-0060] Vélez, J. M. , Berbesí, D. Y. , Cardona, D. , Segura, A. M. , & Ordóñez, J. E. (2012). Validación de escalas abreviadas de zarit para la medición de síndrome del cuidador primario del adulto mayor en Medellín. Atención Primaria, 44(7), 411–416. 10.1016/j.aprim.2011.09.007 22055916 PMC7025946

[nop2732-bib-0061] Vellone, E. , Biagioli, V. , Durante, A. , Buck, H. , Lovino, P. , Colaceci, S. , & Petruzzo, A. (2020). The influence of caregiver preparedness on caregiver contributions to self‐care in heart failure and the mediating role of caregiver confidence. Journal of Cardiovascular Nursing, 35(3), 1–10. 10.1097/JCN.0000000000000632 32084078

[nop2732-bib-0062] World Medical Association Declaration of Helsinki (2013). Ethical principles for medical research involving human subjects. Journal of the American Medical Association, 310(20), 2191–2194. 10.1001/jama.2013.281053 24141714

[nop2732-bib-0063] Yang, C. T. , Liu, H. Y. , & Shyu, Y. I. (2014). Dyadic relational resources and role strain in family caregivers of persons living with dementia at home: A cross‐sectional survey. International Journal of Nursing Studies, 51(4), 593–602. 10.1016/j.ijnurstu.2013.09.001 24083977

[nop2732-bib-0064] Zarit, S.H. , Reever, K.E. , & Bach‐Peterson, J. (1980). Relatives of the Impaired Elderly: Correlates of Feelings of Burden. The Gerontologist, 20(6), 649–655. 10.1093/geront/20.6.649 7203086

